# Koala retroviruses: characterization and impact on the life of koalas

**DOI:** 10.1186/1742-4690-10-108

**Published:** 2013-10-23

**Authors:** Joachim Denner, Paul R Young

**Affiliations:** 1 Robert Koch Institute, Berlin, Germany; 2 School of Chemistry & Molecular Biosciences, University of Queensland, St. Lucia, Brisbane, Australia

**Keywords:** Gammaretroviruses, Koala retrovirus, Lymphoma, Immunodeficiency

## Abstract

Koala retroviruses (KoRV) have been isolated from wild and captive koalas in Australia as well as from koala populations held in zoos in other countries. They are members of the genus *Gammaretrovirus,* are most closely related to gibbon ape leukemia virus (GaLV), feline leukemia virus (FeLV) and porcine endogenous retrovirus (PERV) and are likely the result of a relatively recent trans-species transmission from rodents or bats. The first KoRV to be isolated, KoRV-A, is widely distributed in the koala population in both integrated endogenous and infectious exogenous forms with evidence from museum specimens older than 150 years, indicating a relatively long engagement with the koala population. More recently, additional subtypes of KoRV that are not endogenized have been identified based on sequence differences and host cell receptor specificity (KoRV-B and KoRV-J). A specific association with fatal lymphoma and leukemia has been recently suggested for KoRV-B. In addition, it has been proposed that the high viral loads found in many animals may lead to immunomodulation resulting in a higher incidence of diseases such as chlamydiosis. Although the molecular basis of this immunomodulation is still unclear, purified KoRV particles and a peptide corresponding to a highly conserved domain in the envelope protein have been shown to modulate cytokine expression *in vitro,* similar to that induced by other gammaretroviruses. While much is still to be learned, KoRV induced lymphoma/leukemia and opportunistic disease arising as a consequence of immunomodulation are likely to play an important role in the stability of koala populations both in the wild and in captivity.

## Review

### Distribution of KoRVs

When an increasing incidence of lymphoma and leukemia was recognized in koalas [1], the question was raised whether this may be associated with a retrovirus given similar disease consequences in mice (murine leukemia virus, MuLV) [2,3], cats (FeLV) [4,5], gibbon apes (gibbon ape leukemia virus, GaLV) [6], and humans (human T cell leukemia virus, HTLV) [7]. MuLV, FeLV and KoRV are gammaretroviruses, HTLV is a deltaretrovirus and they all belong to the subfamily *Orthoretrovirinae* of the family *Retroviridae*. Retroviruses use the viral enzyme reverse transcriptase to produce a double-stranded DNA copy of the genomic RNA, which is integrated into the genome of the target cell. This integrated form of the viral genome is referred to as a provirus. At present, three KoRVs have been described, KoRV-A, KoRV-B and KoRV-J [8-10]. The origin of the KoRVs is still unclear, with a high probability that they are the result of a trans-species transmission from rodents or bats, since closely related gammaretroviruses have been found in South Eastern Asian mice [11,12] and bats [13,14]. It is likely that KoRV-A and GaLV, an exogenous retrovirus inducing leukemia in gibbons and using the orthogolous receptor, the sodium-dependent phosphate transporter Pit-1, have the same origin [8]. Of note, the lentiviruses HIV-1 and HIV-2 are also the result of a trans-species transmission [15,16]. KoRV-B and KoRV-J have been shown to encode an envelope gene with an altered receptor binding domain (RBD) resulting in the use of an alternative receptor, the thiamine transport protein 1 (THTR1) [9,10]. KoRV-A has been isolated from koalas in Australia [8,17] and in Japanese [18,19] and German [20] zoos. Retrovirus particles have also been described in animals at the San Diego zoo in the USA [21]. KoRV-B was isolated from animals in the Los Angeles zoo but not from those at the San Diego zoo [9]. KoRV subtypes with modifications to the RBD have also been found in animals from the Duisburg and Antwerpen zoos in Europe as well as from wild koalas in Australia (our unpublished data). KoRV-A sequences have also been found in preserved pelts from animals kept in museums around the world [22]. KoRV-J was first isolated from a koala held in the Kobe Municipal Oji Zoo [18]. Later, 51 animals reared in 9 different Japanese zoos were investigated using differential PCR. 68% of the koalas sourced from northern Australia were positive for KoRV-J, whereas none of the animals sourced from the state of Victoria in the south of Australia were positive for KoRV-J [10]. In a first study investigating the prevalence of KoRV-A in the wild in Australia, a high number of infected animals were found in the North, whereas some animals in the South, especially on Kangaroo island were found to be negative [23]. In a later study investigating a larger number of animals, 100% of koalas in Queensland and New South Wales were again shown to be carrying the virus while 14.8% (24 from 162) of the animals on Kangaroo Island were found to be positive, suggesting recent spread of the virus in this isolated koala population [24]. Subsequent field studies have indicated this figure may be as high as 30-35% (our unpublished data). The prevalence data must also be viewed in the context of wild koala populations that have undergone significant upheaval in the past. This is particularly the case for those populations in southern Australia that were hunted to near extinction in the late 1800s and early 1900s. Many of these areas were subsequently repopulated by the translocation of koalas from other localities but usually employing a relatively small number of animals, resulting in a drastic loss of genetic diversity [25].

## Endogenization of KoRV-A

Retroviruses are divided into exogenous and endogenous forms. Exogenous retroviruses such as the human immunodeficiency virus HIV-1 are transmitted horizontally through infection of their specific somatic target cells (CD4^+^ T lymphocytes in the case of HIV-1). However when a retrovirus infects germ cells, sperm cells or oocytes, the integrated DNA proviruses become a fixture of the genome of all cells of the developing organism. These are called endogenous retroviruses and will be transmitted vertically, inherited along with all other cellular genes [26]. While KoRV-B and KoRV-J appear to be exogenous retroviruses given that less than 1 copy per cell has been detected in tissues from infected animals, KoRV-A has been found in multiple proviral copies in the genome of every cell, including sperm cells of infected animals examined in northern Australia [23]. Its presence in sperm cells along with a host genome integration profile that appears identical in all tissues examined from each individual, indicates that it is endogenous in these populations [23]. However KoRV is not present in many of the koalas sampled across southern Australia [24] suggesting that it may currently be in the process of endogenizing the genome of this species. This unique situation presents an exciting opportunity to study the dynamic process of retroviral endogenization of a wild population in real-time.

## Biological properties of KoRV

**Figure 1:**
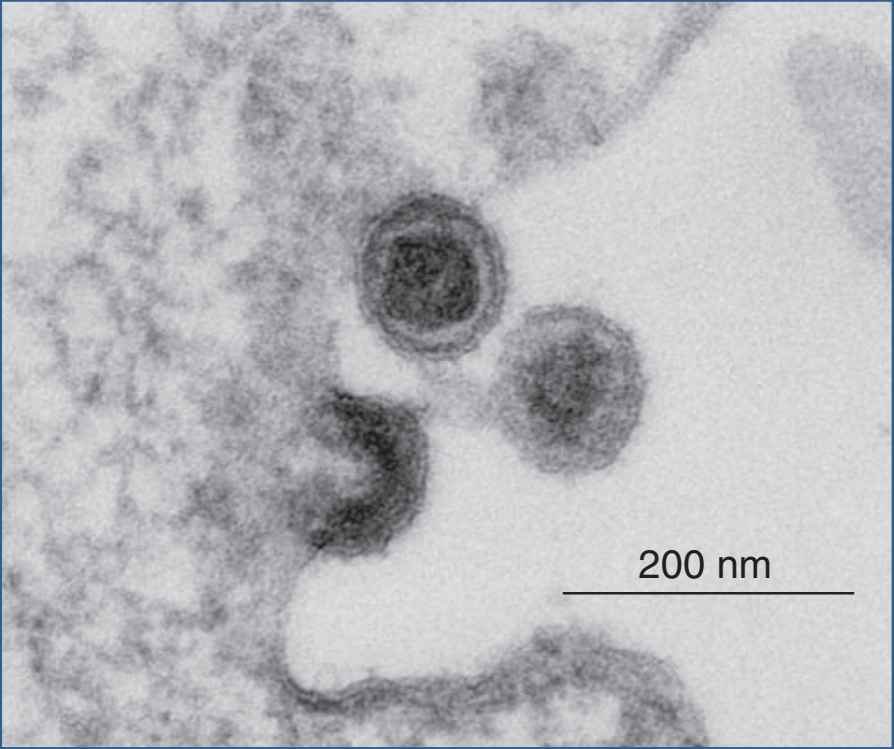
Electron microscopy of KoRV grown in human cells (Holland, Laue, Robert Koch Institute, Berlin).

**Figure 2:**
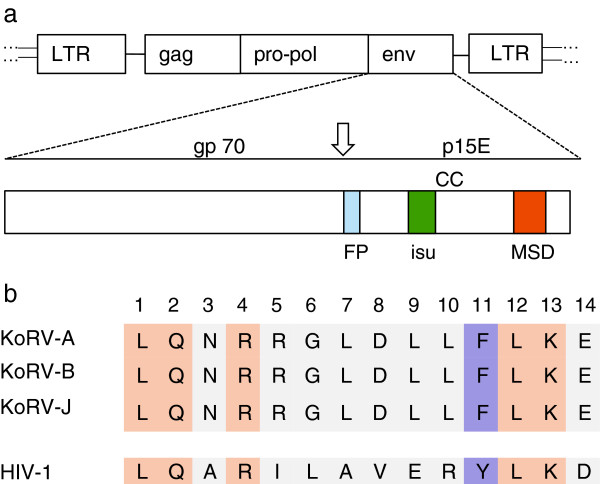
**Schematic presentation of the integrated provirus of KoRV, structure of the envelope protein and sequence of the immunosuppressive domain. a**, LTR, long terminal repeat; gag, group-specific antigen; pro-pol, protease-polymerase; env, envelope protein, FP, fusion peptide; isu, immunosuppressive domain; MSD, membrane spanning domain; CC, cysteine-cysteine loop. The arrow indicated the cleavage site between the surface (gp70) and the transmembrane (p15E) envelope protein. **b**, comparison of the sequence of the immunosuppressive domain of three KoRV subtypes and HIV-1. Identical amino acids are in orange, conservative exchanges in lilac.

The KoRVs have the typical morphology, size (KoRV-A, Figure 1) and genome organization (Figure 2) of the gammaretroviruses. Like all retroviruses they encode a reverse transcriptase and structural proteins including the main core protein p27Gag and the envelope proteins gp70 and p15E [20]. Most studies to date have focused on KoRV-A which has been shown to infect cells of different species (polytropic virus) *in vitro* including rat, human, feline and mink but not mouse cells [10,19,20,27]. It was shown to infect rats *in vivo*, but it remains unclear whether it is pathogenic in rats [20]. KoRV-B also infects a wide range of cells from different species including human [9]. Using pseudotyped KoRV-J, infection of human and cat cells was observed, but not of rat and mouse cells [10]. Infection *in vitro* does not automatically mean that these viruses can infect *in vivo* and give rise to a zoonosis. For example, when the host range of a closely related gammaretrovirus, PERV, was investigated, no infection was observed in humans, primates or other species when transplanting pig cells or injecting concentrated virus, despite the fact that cells of all species with the exception of mice could be infected *in vitro* (for review see [28]).

KoRV-A, -B and -J differ in the sequence of their RBD on the envelope protein gp70, which determines their differential receptor use. In addition, KoRV-B has four repeats of a sequence in the U3 region of the long terminal repeat (LTR) that in KoRV-A is found as a single copy [9]. The U3 region influences gene expression with similar sequence multimerizations in the LTR of MuLV, FeLV and PERV [29-31]. Increased numbers of repeats correlate with increased replicative potential and hence higher virus titers. The sequence differences in the RBD of KoRV-A, KoRV-B and KoRV-J comprise substantial insertion/deletions suggesting that they may be the result of recombination between KoRV-A and another endogenous sequence yet to be identified.

## Diseases associated with a KoRV infection

Retroviruses are known to induce tumors and/or immunodeficiency [3]. In some cases retroviruses are non-pathogenic, for example the foamy viruses [32,33], while others, such as MuLV [2,3], FeLV [4,5], GaLV [6], and HTLV [7] induce leukemia in the corresponding host. In addition, induced immunodeficiency often results in the onset of disease associated with opportunistic infections. Chlamydiosis is a major disease of koalas in both captivity and the wild [2,34], a disease also commonly associated with FIV (feline immunodeficiency virus) infections in cats [35] and HIV infections in humans [36]. In general, the diseases associated with FeLV infection in cats and KoRV infections in koalas are very similar (see list of Diseases reported in both FeLV-infected cats and KoRV-infected koalas and references [1,4,5,8,17,21,34]). Whereas only 5 to 10% of FeLV-infected cats suffer from leukemia and lymphoma, more than 65% of them die from opportunistic infection based on an underlying immunodeficiency [4,5].

Diseases reported in both FeLV-infected cats and KoRV-infected koalas

Lymphoma

Leukaemia

Anemia

Mesothelioma

Craniofacial tumors

Chlamydiosis

Rhinitis/Pneumonia

Stomatitis

Gingivitis

Crytococcosis

Toxoplasmosis

It is important to note that the virus load in the blood of KoRV positive koalas can be extremely high, reaching >10^9^ genome equivalents/ml in some animals [37]. Virus load has been found to correlate with disease progression, a correlation typical for other retrovirus infections [38]. The higher the virus load the higher the probability of insertional mutagenesis, one possible reason for tumor induction. It will also be important to learn whether particular KoRV subtypes are more associated with tumor development than others. Preliminary evidence suggests such an association for KoRV-B [9]. In addition, the higher the virus load, the higher the levels of the immunomodulating transmembrane envelope (TM) protein p15E, implicated as a key mediator of immunodeficiency (see below).

## Does KoRV induce immunodeficiency?

Retroviruses are well known to induce immunodeficiency [3-5,39-42] and it is interesting to note that FeLV-infected cats as well as HIV-1-infected humans are characterized by a decrease in the number of CD4^+^ cells [43]. At present it is unknown whether KoRV infection alters the equivalent CD4^+^ cell count of infected koalas or indeed any other immunological marker. Immunological studies in koalas have been challenging given the absence of readily available reagents, however this is rapidly changing and the next few years should see a wealth of immunological measurements appearing in the literature.

The molecular mechanisms by which retroviruses induce immunodeficiency are still not completely understood. But there is accumulating evidence that the viral transmembrane (TM) protein, p15E, of all gammaretroviruses including KoRV is involved. All retroviral TM proteins contain a highly conserved sequence, referred to as the immunosuppressive (isu) domain (Figure 2). Viral particles and recombinant or native viral TM proteins of HIV-1 [44-47], human endogenous retrovirus HERV-K [48], PERV [40,49] and KoRV [20] have been shown to inhibit lymphocyte activation by mitogens and modulate cytokine expression in peripheral blood mononuclear cells (PBMCs). Single mutations in the isu domain of HIV-1 gp41 abrogated the immunosuppressive activity of the molecule and immunization with the mutated gp41 resulted in better antibody responses when compared with wild-type gp41 immunization [47]. The interleukins IL-10 and IL-6 were shown to be elevated and molecules involved in innate immunity were down-regulated. Synthetic peptides corresponding to the isu domains were able to inhibit lymphocyte activation and to modulate gene expression in the same manner as the TM protein and whole viruses [50-53].

Retroviral TM proteins have also been shown to be immunosuppressive *in vivo*. Transformed cells that do not naturally develop tumors when injected into immunocompetent mice but do form tumors in immunocompromized mice, could be converted into tumor forming cells even in immunocompetent mice when TM proteins were expressed on their surface [41,42,54-56]. The expressed TM proteins not only prevented rejection of the transplanted cells, but also inhibited the humoral immune response as well as NK and CD8^+^ cells in the recipient animal [56,57]. The sequence of the isu domain is identical in all KoRV subtypes so far sequenced (Figure 2) and it would be surprising if the high viral loads carried by KoRV positive koalas throughout their lives did not have some impact on immune function.

## Impact on the life of koalas

As noted above, many gammaretroviruses are known to cause neoplastic and immunosuppressive diseases in their respective hosts [2-5]. The fact that koalas suffering from lymphoma/leukemia have been shown to have higher levels of circulating KoRV viraemia [37] and that koalas also suffer from an unusually high incidence of chlamydiosis, a disease associated with immunosuppression induced by gammaretroviruses in other species [35,36], suggests that KoRV is no exception. These consequences of higher levels of KoRV load are most likely the result of an increase in the probability of mutational insertion and/or recombination for the induction of tumors and increased immunodeficiency. Therefore it is not surprising that populations with low levels of KoRV appear to have a correspondingly lower incidence of diseases than those with higher viral loads [37]. Although there is still no direct causal evidence for KoRV induced hematopoetic neoplasia and immunosuppression, the data published to date and comparison with other retroviral infections including HIV-1 strengthen the case for an association between KoRV and disease in koalas.

There are many challenges to the life of a koala in the wild. With an ever-expanding human population, many koala populations live in close urban association. Consequent ongoing habitat destruction, construction of roads that transect koala habitats, and attacks from domestic pets such as dogs pose an ever-increasing threat to population stability. However disease poses an additional and significant threat to koalas. Of particular concern are the increasing rates of KoRV infection seen in some populations [23,24] suggesting that all animals may ultimately become infected. If the virus becomes endogenous in all animals this process is irreversible and the virus will be transmitted to all subsequent generations.

## Strategies for preventing infection and disease

To prevent the further spread of KoRV in the koala population several strategies are potentially available; (i) The isolation or quarantine of uninfected koalas to prevent contact with infected animals. However this would only be possible for some groups of southern koala populations, since all populations in the north are already carrying the virus. (ii) Antiretroviral drug treatment for infected koalas. However the number of such drugs available for gammaretroviruses is limited. Susceptibility *in vitro* has been shown for the triphosphorylated nucleoside analog of zidovudine (AZT), ddGTP and to a lesser extent to ddTTP but almost no susceptibility to the non-nucleoside RT inhibitors was observed when activity against the related PERV was tested [58,59]. Even if a suitable anti-retroviral could be identified, treatment of koalas in captivity may be possible but the logistics would likely mean little or no impact on wild koala populations. (iii) Perhaps one of the most likely intervention strategies could be opportunistic vaccination. Thousands of animals are taken into care each year throughout Australia and then released back into the wild. These could be vaccinated while in care. In contrast to HIV-1, where all attempts to generate an effective vaccine have failed, protecting vaccines against gammaretroviruses have been successfully generated. The best example is FeLV where commercial vaccines exist which protect cats from virus replication and disease, however not from infection [43,60]. Better immunization strategies and antigens may lead to complete protection from infection. In addition to the vaccines against FeLV, experimental vaccines have been developed for MuLV. These approaches have included killed virus [61], subunit vaccines [62,63], and live attenuated viruses [64,65]. Immunization with the TM proteins of PERV [66-68], FeLV [60,69,70] and KoRV [20 and unpublished data] induce effective neutralizing antibodies mainly targeting epitopes in the membrane proximal external region (MPER) of the TM protein. When immunizing with a combination of the TM protein and the surface envelope protein (SU) gp70 of FeLV [71,72] and PERV [71,73], higher titers of neutralizing antibodies were induced. In the case of FeLV it was shown that these vaccinations protected cats from infection *in vivo*[60].

## Outlook

Based on immunization experiments with gammaretroviruses closely related to KoRV, a similar effort to induce neutralizing antibodies against KoRV should be undertaken. Prevention of infection with KoRV (preventive vaccination) or decreasing the virus load (therapeutic treatment) in already infected animals could prevent or reduce KoRV-induced immunomodulation and therefore also protect animals from infection with chlamydia and other opportunistic infections. While this approach could offer significant additional management options for koalas in captivity, the challenge would be in the effective delivery of such a vaccine to a wild population dispersed over a broad geographic range.

## Conclusion

The spread of a growing number of newly recognized KoRV genotypes in both wild and captive koalas has the potential to significantly impact the health of many koala populations. The long-term consequences to the species of these endogenizing elements are currently unknown. Koalas may eventually evolve and adapt to this genomic intruder, just as many other vertebrates, including humans have accommodated the array of retroviral elements that make up their genomes. However in the meantime, this relatively new and ongoing engagement between a group of retroviruses and their host is resulting in the appearance in the koala population of the same spectrum of diseases typically associated with many exogenous retroviral infections. While there are significant, and perhaps insurmountable logistical challenges in proposing any intervention strategies for many wild koala populations, this may be an option for koalas in captivity and some selected isolated wild koala populations. Both prevention and treatment strategies could be employed. An effective vaccine or therapeutic intervention would reduce virus load which in turn would likely reduce the induction of lymphoma/leukemia and the number and severity of opportunistic diseases arising as a consequence of immunomodulation. A reduction in virus load is also likely to reduce the probability of virus transmission. As for many other retrovirus examples, a preventive vaccine could be the best way to prevent further spread of the virus infection. Regardless of whether any of these strategies for control are feasible, the ongoing process of retroviral endogenization of the koala genome presents, for the first time an ideal opportunity to study this process in a wild population in real-time.

## Competing interests

The authors declare that they have no competing interests.

## Authors’ contribution

Both authors contributed equally to this review. Both authors read and approved the final manuscript.
